# Antibacterial Activity of Juglone against *Staphylococcus aureus*: From Apparent to Proteomic

**DOI:** 10.3390/ijms17060965

**Published:** 2016-06-18

**Authors:** Jiayi Wang, Yuhuan Cheng, Rina Wu, Donghua Jiang, Bing Bai, Dehong Tan, Tingcai Yan, Xiyun Sun, Qi Zhang, Zhaoxia Wu

**Affiliations:** College of Food Science, Shenyang Agricultural University, 120 Dongling Rd., Shenyang 110866, China; jiayiwangsyau@syau.edu.cn (J.W.); 15802496575@163.com (Y.C.); wrn6956@163.com (R.W.); donghuajiang@syau.edu.cn (D.J.); baibingsyau@syau.edu.cn (B.B.); tandehongsy@126.com (D.T.); ytc126127@163.com (T.Y.); sun_xiyun@syau.edu.cn (X.S.); xiaodi1993@163.com (Q.Z.)

**Keywords:** juglone, *Staphylococcus aureus*, iTRAQ, antibacterial activity

## Abstract

The proportion of foodborne disease caused by pathogenic microorganisms is rising worldwide, with staphylococcal food poisoning being one of the main causes of this increase. Juglone is a plant-derived 1,4-naphthoquinone with confirmed antibacterial and antitumor activities. However, the specific mechanism underlying its antibacterial activity against *Staphylococcus aureus* remains unclear. To elucidate the mechanism underlying its antibacterial activity, isobaric tags for relative and absolute quantitation methods of quantitative proteomics were applied for analysis of the 53 proteins that were differentially expressed after treatment with juglone. Combined with verification experiments, such as detection of changes in DNA and RNA content and quantification of oxidative damage, our results suggested that juglone effectively increased the protein expression of oxidoreductase and created a peroxidative environment within the cell, significantly reducing cell wall formation and increasing membrane permeability. We hypothesize that juglone binds to DNA and reduces DNA transcription and replication directly. This is the first study to adopt a proteomic approach to investigate the antibacterial mechanism of juglone.

## 1. Introduction

Food-borne diseases (FBD) are defined by the World Health Organization (WHO) as “diseases of infectious or toxic nature caused by, or thought to be caused by, the consumption of food or water [1].” Numerous food-borne diseases, including staphylococcal food poisoning (SFP), are caused by ingestion of microbial and plant toxins [2], and SFP is mainly caused by *Staphylococcus aureus* [3]. SFP causes various symptoms, including copious vomiting, diarrhea, abdominal pain, and nausea [4], owing to the production of staphylococcal enterotoxins (SEs). Although approximately 22 SEs [3] are known, only a few of these proteins, such as SEA and SEB, are related to FBD [5]. Hence, it is imperative to control the spread of *S. aureus* to ensure food safety.

**Figure 1:**
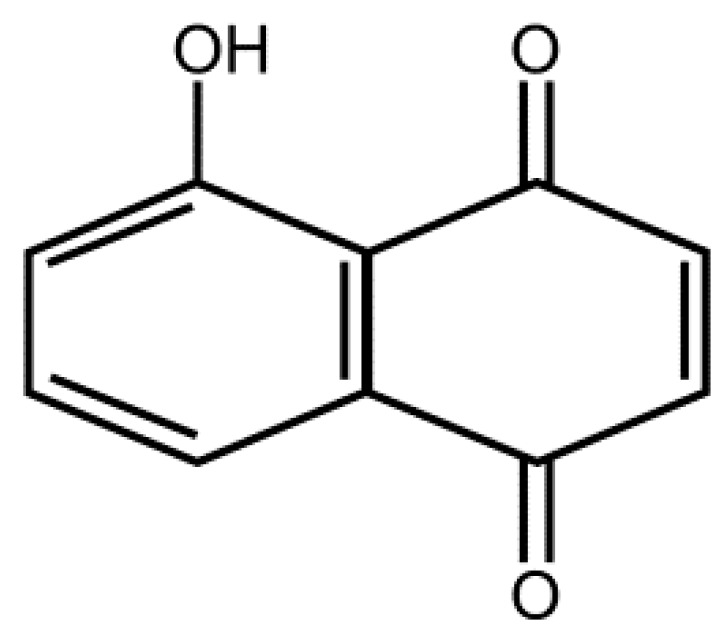
Molecular structure of juglone.

Natural products with pharmacological properties often exhibit broad-spectrum antibacterial activity and have unique advantages. Naphthoquinones, such as juglone, lawsone, plumbagin, and lapachol, are natural products with high antibacterial activity. In particular, juglone (5-hydroxy-1,4-naphthoquinone) (Figure 1) has been used for centuries in folk medicines to cure acne, allergies, gastrointestinal disorders, intestinal parasitosis, cancer, fungal infections, bacterial infections, and viral infections [6]. Our previous study revealed that juglone shows antibacterial activity against *S. aureus*, *Escherichia coli*, *Bacillus subtilis*, *Penicillium* sp., *Aspergillus* sp., and *Hansenula* sp. [7]. According to previous studies, naphthoquinones exert their antimicrobial, antiparasitic, and cytotoxic activities via several mechanisms, including inhibition of electron transport, uncoupling effects during oxidative phosphorylation, intercalation of agents into the DNA double helix, reduction of alkylating properties of biomolecules, and production of reactive oxygen species (ROS) under aerobic conditions [6]. However, in recent years, most investigations of juglone have focused on its antitumor activity and the related molecular mechanisms. However, a more in-depth understanding of how juglone acts against bacteria, especially *S. aureus*, is still lacking. Therefore, to elucidate the possible mechanism of action of juglone on *S. aureus*, we adopted a proteomic approach owing to its suitability for high-volume data processing. Furthermore, proteomics could help reveal changes in the whole proteome post juglone-treatment, in contrast to currently used methods such as superoxide dismutase activity assays, malondialdehyde evaluation, and electron microscopic analysis. In this study, we investigated the proteomic alterations in *S. aureus* following treatment with juglone using isobaric tags for relative and absolute quantitation (iTRAQ) technology, and then identified the altered proteins to reveal the antibacterial mechanism of juglone.

## 2. Results and Discussion

### 2.1. iTRAQ Analysis of the Proteome after Treatment with Juglone

Compared to the initially popular gel-based proteomic technology, MS-based proteomic analyses are now widely used because of their high-throughput capacity, repeatability, and high success rate for protein identification. In the current study, normal *S. aureus* and *S. aureus* treated with juglone for 2 h were collected for protein extraction, digestion, and iTRAQ labeling during the exponential growth phase. As a mainstream MS-based proteomics technology, iTRAQ can provide multiplexing of up to 8-plex isobaric tags including a reporter group, a balance group, and a peptide-reactive group. Once the isobaric tags have reacted with the proteolytic peptides, the balance group is removed to identify the differentially expressed peptides at the second mass spectrometry (MS2) level. In a search using the Mascot 2.2 program, we identified 9834 unique peptides (FDR ≤ 0.1), corresponding to 1379 protein groups including 1376 proteins that were quantified by Proteome Discoverer 1.4 in each channel. In total, the expression levels of 53 proteins were shown to be significantly different (>1.2-fold change, *p* < 0.05) between treated and untreated cells. Among these proteins, 22 were up-regulated and 31 were down-regulated in the treated cells compared to the untreated cells.

### 2.2. Functional Annotation Analysis of Proteomic Differences

To determine the function of the 53 differentially expressed proteins, we performed annotation analysis using Blast2Go. The proteins were grouped into six categories (Table 1): oxidative damage, DNA replication and transcription, protein synthesis, stress response, cell wall synthesis and cell division, and membrane permeability.

### 2.3. Upregulation of Glyoxalase, Potassium Uptake Protein, and Nitroreductase

**Figure 2:**
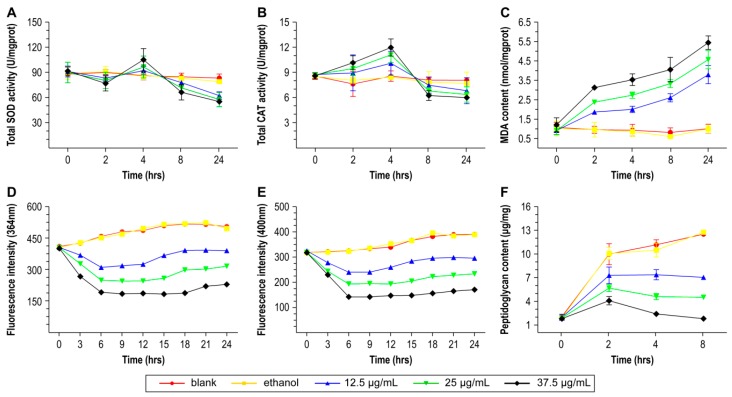
Indicators of damage to *Staphylococcus aureus* after treatment with juglone. **A**–**C** show changes in total superoxide dismutase (SOD) activity, total catalase (CAT) activity, and malondialdehyde (MDA) content, respectively; **D**,**E** show changes in DNA and RNA fluorescence intensity, detected at 364 nm and 400 nm; **F** shows changes in the peptidoglycan content. Error bar indicates SEM.

After 2 h of treatment, upregulation of the proteins glyoxalase, potassium uptake protein, and nitroreductase, which belong to the oxidoreductase protein family, was induced by juglone, and resulted in subsequent cell collapse. Additionally, the formation of superoxide radicals is often triggered by metal ions (mostly iron, but also copper, cobalt, and titanium), which are defined as cofactors. These transition metal ions can be transferred from superoxide radicals to hydroxyl free radicals, which are the strongest known oxidizing agents. Such activity may be suggested by the observed up-regulation of serine-rich-repeat-containing protein, which has a calcium-binding function. As shown in Figure 2a, in the groups treated with 12.5, 25, and 37.5 µg/mL juglone, the superoxide dismutase (SOD) activity decreased before 2 h, from 91.07 to 83.15, 89.81 to 80.34, and 91.59 to 76.79 U/mgprot, respectively. This result suggests that *S. aureus* had not adapted to the excess of superoxide anions before 2 h and consumed the original SOD to generate hydrogen peroxide, resulting in catalase (CAT) activity increasing from 8.72 to 8.91, 8.7 to 9.42, and 8.56 to 10.12 U/mgprot before 2 h, as shown in Figure 2b. At 4 h, the superoxide anion concentration had far exceeded the capacity of SOD, resulting in the decrease observed after 4 h. Correspondingly, CAT was quickly consumed starting at 4 h. Combined with our proteomic results, these results suggest that juglone could accelerate the redox process leading to oxidative damage to *S. aureus*, and that the cell’s own CAT and SOD were not sufficient to cope with the oxidative damage starting at 4 h.

### 2.4. Significance of the Downregulation of Thioredoxin, Threonine Dehydratase, and Ribulose-5-phosphate 3-Epimerase-epimerase

To survive in a peroxidative environment, organisms produce several natural antioxidants, including vitamin C [8], glutathione (GSH) [9], and carotenoids [10], among others. Carotenoids show antioxidative activity based on their ability to trap peroxyl radicals and quench singlet oxygen. Here, 4,4′-diaponeurosporenoate glycosyltransferase, which plays a major role in carotenoid biosynthesis, was down-regulated after treatment with juglone. This result suggested that production of 4,4′-diaponeurosporenoate glycosyltransferase was inhibited by juglone, which resulted in fewer carotenoids in the cells [11]. Moreover, thioredoxin was down-regulated. This protein is a cell redox homeostasis regulator, and its role is to maintain the stability of cellular levels of ROS. Threonine dehydratase was also down-regulated, possibly because of a shortage of iron [12]. Moreover, ribulose-5-phosphate 3-epimerase-epimerase was found to be down-regulated. This molecule in *E. coli* was reported to be rapidly damaged by hydrogen peroxide [13]; therefore, its down-regulation might have been directly caused by hydrogen peroxide. The change in expression levels of these proteins supports the proposition that oxidative damage was the main mechanism of activity against *S. aureus*.

### 2.5. Downregulation of Proteins Related to DNA Replication and Transcription

All proteins related to DNA replication and transcription were down-regulated after treatment with juglone for 2 h. DNA-binding response regulator, family transcriptional regulator, and transcriptional regulator MraZ regulate DNA-dependent transcription, while Queuosine biosynthesis protein plays a major role in the tRNA modification process. Queuine is one of the most radically modified nucleosides known to occur in tRNA [14], and its expression level is regulated by queuine tRNA-ribosyltransferase. Other proteins related to DNA transcription, including uridine kinase and urease accessory protein ureg, participate in guanosine triphosphate (GTP) and cytidine triphosphate (CTP) biosynthesis, which plays a major role in the formation and phosphorylation of RNA. Our results suggest that anaerobic ribonucleoside triphosphate and single-stranded DNA-binding protein, which are associated with DNA replication, were also down-regulated. Similarly, as shown in Figure 2d,e, the fluorescence intensity of DNA and RNA in the treated groups maintained a sustained decreasing trend from 0 to 6 h. It was previously reported that juglone induces scission of isolated DNA by reducing glutathione and Fe (II) ions *in vitro* [15]. In addition, 1,4-naphthoquinones induce oxidative damage to DNA base pairs and accumulation of DNA breaks [16]. The best-known mechanism is intercalation between two base pairs of DNA or RNA through the ring of the polycyclic chromophore of quinone. Anthracyclines, which are quinone compounds, exhibit antitumor activity by forming bonds between positively charged amino sugars and the sugar phosphate backbone of DNA [17]. In this manner, several vital biological processes, such as replication and transcription, are blocked. Thus, based on these experimental results, we hypothesize that juglone binds to DNA and directly causes DNA damage.

### 2.6. Role of Juglone in Stimulating Stress Response in S. aureus

DNA damage and oxidative damage can stimulate the stress response in *S. aureus*. The triglyceride lipase, lipase 1, was also upregulated. Lipase 1 can catalyze use of triacylglycerols (TAG) as a carbon and energy source for survival during starvation conditions [18]. This result suggested that energy metabolism was induced to protect against stress. However, we did not identify upregulation of any proteins related to energy metabolism. This might be attributed to limitations of the database, as 14 of the upregulated proteins could not be annotated and need to be studied further. ATP guanido phosphotransferase and the heat resistance chaperone protein clpB were both upregulated. CtsR-dependent genes were previously shown to be weakly induced in response to oxidative stress; CtsR is a repressor of heat- and stress-specific proteins [19]. However, ATP guanido phosphotransferase acts as a modulator of CtsR repressor activity under oxidative stress [20]. Hence, protein chaperones might be positively regulated by ATP guanido phosphotransferase. Iron-sulfur cluster repair di-iron protein is a di-iron-containing protein involved in repairing iron-sulfur clusters damaged by oxidative and nitrosative stress [21,22]. Chang *et al.* [23] found that iron-sulfur cluster repair di-iron protein could be induced by hydrogen peroxide, which also supports that juglone acts via oxidative damage. Only one protein, single-stranded DNA-binding protein, related to the DNA damage response was found to be upregulated.

### 2.7. Role of Juglone on Protein Synthesis, Cell Wall Formation, and Permeability

In addition, we noted the impact of juglone on protein synthesis. Three structural components of ribosomes, *i.e.*, 50s ribosomal protein l36, 50s ribosomal protein l14 and 50S ribosomal protein L33 2, participate in translation and were down-regulated after treatment with juglone. Proteins associated with cell division, *i.e.*, iron-sulfur cluster repair di-iron protein and transcriptional regulator MraZ, were also down-regulated. These results indicate that protein synthesis and cell division were inhibited after treatment with juglone. Figure 2f shows that in the juglone-treated group, the peptidoglycan content was markedly lower than that in the control group, increasing before 2 h and decreasing starting at 4 h. Accessory sec system glycosylation protein is an *N*-acetyltransferase that is part of the SecA2/SecY2 system for synthesis of serine-rich cell wall proteins, and is up-regulation suggested that the formation of the cell wall, was not inhibited before 2 h of treatment. Similarly, we observed upregulation of N-acetylmuramoyl-l-alanine amidase, an enzyme that catalyzes a chemical reaction that cleaves the link between N-acetylmuramoyl residues and l-amino acid residues in certain cell-wall glycopeptides. These results suggest that cell wall formation was weakly inhibited by 2 h and strong inhibited starting at 4 h. Moreover, two types protein related to cell membrane synthesis, thioredoxin and acetyl-biotin carboxylase subunit, were down-regulated. Thioredoxin participates in metabolism of glycerol ether, which is an important component of cell membranes. Acetyl-biotin carboxylase subunit has acetyl-CoA carboxylase activity, and multi-subunit acetyl-CoA carboxylase can catalyze the first step in fatty acid biosynthesis [24], promoting cell membrane formation. In Figure 2c, the groups treated with 12.5, 25, and 37.5 µg/mL juglone, the malondialdehyde (MDA) content steadily increased from initial 0.9 to final 3.78, 0.89 to 4.55, and 1.19 to 5.43 nmol/mgprot, respectively. These results suggest that cell membrane formation was mainly inhibited through oxidative damage. In addition, potassium uptake protein and chaperone protein clpB, which have cation transmembrane transporter activity, were up-regulated, indicating an increase of permeability. However, gas chromatography analysis (Table 2) showed a decrease in saturated fatty acids (SFA)/unsaturated fatty acids (UFA), from 1.3% to 1.21%, and ultimately to 1.17% as the juglone concentration increased. This result suggests that membrane fluidity was increased [25], resulting in increased permeability, consistent with our proteomic analysis.

In conclusion, this work describes the investigation of the mechanism of action (antibacterial activity) of juglone, a plant-derived 1,4-naphthoquinone. In particular, iTRAQ technology was applied for analysis of the 53 proteins found to be differentially expressed after treatment with juglone, a plant-derived 1,4-naphthoquinone. Combined with verification experiments, the results suggest that oxidative damage was the primary *S. aureus* cell-killing mechanism. In the induction process, juglone up-regulated oxidoreductase, thereby enhancing the redox process and subsequently creating a peroxidative environment in the cell. In addition, juglone significantly decreased cell wall formation, inhibited cell membrane formation, and increased membrane permeability. However, this study had several limitations. Only one strain of ATCC6538 was used because of funding limitations. Juglone may show a different mechanism of action against other species of bacteria (e.g., *Escherichia coli*, *Bacillus subtilis*, *Penicillium* sp., *Aspergillus* sp., and *Hansenula* sp.), and future studies should investigate the effects of juglone on these species. In addition, these data do not comprehensively reveal the antibacterial mechanisms of juglone against *S. aureus*, and have not, for example, identified potential drug targets. Future studies could employ methods such as cocrystallization, AutoDock, and subcellular proteomic analysis to fully reveal the mechanism of action.

## 3. Materials and Methods

### 3.1. Strain and Juglone

*S. aureus* ATCC6538 was purchased from the American Type Culture Collection. The minimal inhibition concentration (MIC) of juglone (Sigma, St Louis, MO, USA) against *S. aureus* is 37.5 µg/mL [7].

### 3.2. Culture Preparation

Juglone (dissolved in anhydrous ethanol) was incubated with *S. aureus* during the exponential growth phase at final concentrations of 0 µg/mL, 12.5 µg/mL, 25 µg/mL, and 37.5 µg/mL in beef extract peptone medium for 24 h at 37 °C, and a control group was incubated with anhydrous ethanol. Each different concentration group included three biological replicates. Cultures were harvested at 12 time points: 0 h, 2 h, 3 h, 4 h, 6 h, 8 h, 9 h, 12 h, 15 h, 18 h, 21 h and 24 h.

### 3.3. Protein Preparation

To quantify changes to the proteome after treatment with juglone, the cultures were incubated with juglone during the exponential growth phase at a final concentration of 18.75 µg/mL for 2 h at 37 °C. Then quartz sand and 1 mL of SDT lysate (4% SDS, 1 mM DTT, 100 mM Tris-HCl, pH 7.6) were added to each group and subjected to 10 rounds of homogenization. The homogenate was sonicated on ice. After 5 min of incubation in boiling water and 5 rounds of further homogenization, the crude extract was then incubated in boiling water for 10 min and clarified by centrifugation at 13,400× *g* for 30 min. The supernatant was filtered through a 0.22-µm membrane, and proteins were quantified using a bicinchoninic acid (BCA) protein assay (Beyotime, Shanghai, China).

### 3.4. Protein Digestion and iTRAQ Labeling

Total protein from each sample was digested using filter-aided proteome preparation (FASP) method as described previously by Wisniewski *et al.* [26], and the peptide mixture was labeled with 8-plex iTRAQ reagent (AB SCIEX, Framingham, MA, USA) according to the manufacturer’s instructions.

### 3.5. Strong Cation-Exchange Chromatography (SCX) Fractionation

To reduce sample complexity, the labeled peptides were dissolved with 2 mL buffer A (10 mM KH_2_PO_4_ in 25% ACN, pH 3.0) and separated by SCX chromatography (GE Healthcare) using a PolySULFOETHYL column (4.6 × 100 mm, 5 µm, 200 Å, PolyLC Inc., Columbia, MD, USA). The elution was performed at a flow rate of 1 mL/min with a gradient of 0%–10% buffer B (500 mM KCl, 10 mM KH_2_PO_4_ in 25% ACN, pH 3.0) for 2 min, 10%–20% buffer B for 25 min, 20%–45% buffer B for 5 min, and 50%–100% buffer B for 5 min. The absorbance wavelength was set at 214 nm, and fractions were collected every 1 min. The collected petides were combined into 6 fractions, desalted using a C18 cartridges (Empore SPE Cartridges C18 (standard density), bed I.D. = 7 mm, volume = 3 mL, Sigma), and concentrated by vacuum centrifugation. Finally, the dried fractions were reconstituted in 40 µL of 0.1% (*v*/*v*) trifluoroacetic acid, and stored at −80 °C for further liquid chromatography electrospray ionization tandem mass spectrometry (LC-ESI-MS/MS) analysis.

### 3.6. LC-ESI-MS/MS

A quantity of 5 μg of peptide mixture was separated by Easy nLC system using a C18-reversed phase column (Easy nLC system, Thermo Scientific Easy Column) (100 mm × 75 μm, 3 μm). The separation was achieved using a linear gradient of buffer B (80% acetonitrile and 0.1% formic acid) at a flow rate of 250 nL/min over 140 min. Q Executive mass spectrometer (Thermo Fisher Scientific, Waltham, MA, USA) acquired data was filtered by choosing the most abundant precursor ions, with a range of 300–1800 *m*/*z* for higher-energy collisional dissociation (HCD) fragmentation. The target value was determined basing on predictive Automatic Gain Control (pAGC). Dynamic exclusion was used with 1 min. Survey scans were set as a resolution of 70,000 at *m/z* 200, and 17,500 at *m*/*z* 200 resolution for HCD spectra. The normalized collision energy was 30 eV and the underfill ratio, which specifies the minimum percentage of the target value likely to be reached at maximum fill time, was defined as 0.1%. The instrument was run with mode enabled peptide recognition.

### 3.7. Database Searching and Data Analysis

MS/MS spectra were identified by using the Mascot engine (Matrix Science, London, UK; version 2.2) to search the UniProt *S. aureus* NCTC8325 database (downloaded May 2015, 2896 sequences). Proteome Discover was used to identify the proteins. The following detailed options were selected: peptide mass tolerance = ±20 ppm; MS/MS tolerance = 0.1 Da; enzyme = trypsin, missed cleavage = 2; fixed modification: carbamidomethyl (C), iTRAQ 8-plex (K), iTRAQ 8-plex (N-term); variable modification: oxidation (M); decoy database pattern = reverse.

The Mascot search results for each SCX elution were further processed using Proteomics Tools (Matrix Science, Boston, MA, USA) (version 3.05), which includes the programs BuildSummary, Isobaric Labeling Multiple File Distiller, and Identified Protein iTRAQ Statistic Builder. The BuildSummary program was used for assembling protein identifications based on a target-decoy search in shotgun proteomics. All reported data were based on 99% confidence in protein identification as determined by the peptide false discovery rate (FDR) ≤1% [27].

The programs Isobaric Labeling Multiple File Distiller and Identified Protein iTRAQ Statistic Builder were used to calculate protein ratios. The final protein ratios were then normalized by the median average protein ratio for unequal amounts of the labeled samples.

### 3.8. Statistical and Bioinformatics Analysis

Comparisons between treatment and control groups were performed using *t*-tests. Differentially expressed proteins were classified using the following scale: more than 1.2-fold (*p* < 0.05) or less than 0.83-fold (*p* < 0.05) [28].

Gene ontology (GO) analysis was performed using Blast2Go version 3.0; the detailed procedure was described by Stefan *et al.* [29].

### 3.9. Detection of Changes in DNA and RNA Content

Culture samples (2 mL, collected at 0 h, 3 h, 6 h, 9 h, 12 h, 15 h, 18 h, 21 h, and 24 h) were centrifuged at 4000× *g* for 10 min. The precipitates were washed with PBS buffer three times using centrifugation at 4000× *g* for 5 min, and then dissolved in sterile water to prepare 1-mL bacterial suspensions. Next, 0.3 mL of each bacterial suspension was mixed with 0.9 mL of DAPI, and absorbance was detected at 364 nm (DNA) and 400 nm (RNA).

### 3.10. Peptidoglycan Content Determination

Glucosamine standard solution (50 μg/mL) was aliquoted into 6 burets (0 mL, 0.5 mL, 1.0 mL, 1.5 mL, 2.0 mL, and 2.5 mL), and 1 mL of acetylacetone was added, along with sterile water to 6 mL. After 30 min of incubation in boiling water, 4 mL of anhydrous ethanol and 1 mL of Ehrlich’s reagent were added to the cooled solution. The resulting mixture was then incubated in 60 °C water for 1 h, and absorbance was detected at 530 nm. Absorbance values were used to construct a standard curve.

Then, 10-mL culture samples (0 h, 2 h, 4 h, and 8 h) were subjected to five repeated cycles of freezing and thawing, and sonicated on ice. The resulting mixture was centrifuged at 1000× *g* for 10 min, and the supernatant was centrifuged at 10,000× *g* for 20 min. After three rounds of washing, the crude peptidoglycan pellets were collected, and 2 mg of dried material was added to 1.5 mL of hydrochloric acid (6 mol/L) and incubated in boiling water for 1 h. Sodium hydroxide was added to the cooled solution to reach pH 7, and sterile water was added to a total volume of 10 mL. Finally, the concentration of peptidoglycan was calculated as described in the previous paragraph, and the values were used to construct a standard curve, with the following modification: 2 mL of the mixture was used rather than the glucosamine standard solution.

### 3.11. Determination of the Extent of Oxidative Damage

Cultures (0 h, 2 h, 4 h, 8 h, and 24 h) were centrifuged at 4000× *g* for 10 min, and then washed with PBS three times. After resuspension in 2 mL of normal saline (NS), the mixture was sonicated on ice and centrifuged (12,000× *g*, 20 min, 4 °C) and the supernatant was collected. Concentrations of SOD, CAT, MDA, and proteins were determined according to the manufacturer’s instructions (Beyotime, Shanghai, China).

### 3.12. Phospholipid Extraction

Cultures (2 h) were centrifuged at 10,000× *g* for 5 min, and the cell pellets were washed with PBS three times. After resuspension in NS, samples were sonicated on ice and two volumes of chloroform-methanol (2:1, *v*/*v*) were added; the mixture was vortexed for a further 30 min. After centrifugation (2,500× *g*, 10 min), the lower phase was transferred to a new tube. The mixture was added into a quarter volume of methanol-water (1:1, *v*/*v*), and the lower phase was concentrated to 1 mL. Finally, the concentrate was vacuum-dried and stored until use.

### 3.13. Gas Chromatography Analysis of Fatty Acids

Dried phospholipids (0.02 g) were added to 0.5 mL of benzene–petroleum ether (1:1, *V*/*V*), and then 1.5 mL of potassium hydroxide-methanol (0.4 mol/L) was added. After incubation in 50 °C water for 15 min, hexane was added to the cooled solution to a total volume of 10 mL. Samples were vortexed, and the lower phase was collected for gas chromatography analysis. The following parameters were used for gas chromatography: a 30 m × 0.32 mm × 0.25 μm cp-Sil 19 CB silica capillary column was used; the temperature program ramped from 40 °C (2 min hold) to 260 °C (1 min hold) at 3 °C per minute; the injector and detector were held at 250 °C and 300 °C, respectively, with nitrogen used as the carrier gas; the flow rate was 2 mL/min, and the injection volume and split ratio were 1 μL and 35:1 respectively. Finally, a standard mixture that included 37 types of fatty acid methyl esters (Sigma, St. Louis, MO, USA) was used to determine the relative content of fatty acids through area normalization processing.

## Figures and Tables

**Table 1:** Proteins that showed differential expression following treatment of *Staphylococcus aureus* with juglone.

Accession	Protein Name	Gene id	Unique Peptides	Total Peptides	*M*w (kDa)	Fold Change	*p*-Value
*Oxidative damage*							
Q2FVA5	glyoxalase	SAOUHSC_02828	5	5	30.00	1.824	0.012
Q2FZH1	potassium uptake protein	SAOUHSC_01034	2	2	24.30	1.401	0.037
Q2FUW1	serine rich repeat containing protein	sasA	1	1	227.90	1.406	0.027
Q2FWN0	nitroreductase	SAOUHSC_02258	4	4	24.00	1.203	0.005
Q2FZD2	thioredoxin	trxA	9	9	11.40	0.654	0.001
Q2G000	thioredoxin	SAOUHSC_00834	6	6	12.10	0.758	0.046
Q2FXI6	thioredoxin	SAOUHSC_01860	8	8	11.80	0.781	0.003
Q2FZ62	ribulose-phosphate 3-epimerase	SAOUHSC_01189	2	2	23.60	0.750	0.038
Q2FWJ9	threonine dehydratase biosynthetic	ilvA	3	3	46.90	0.751	0.016
Q2FV58	4,4′-diaponeurosporenoate glycosyltransferase	crtQ	2	2	42.50	0.782	0.016
*DNA replication and transcription*							
Q2FWH6	DNA-binding response regulator	SAOUHSC_02315	2	2	26.50	0.787	0.034
Q2FXW6	uridine kinase	Udk	3	3	23.50	0.798	0.022
Q2FV02	anaerobic ribonucleoside-triphosphate	SAOUHSC_02942	3	3	70.40	0.711	0.030
Q2G112	single-stranded DNA-binding protein	SAOUHSC_00349	7	7	18.50	0.816	0.014
Q2FZ97	transcriptional regulator MraZ	mraZ	3	3	17.20	0.819	0.006
Q2FV69	family transcriptional regulator	SAOUHSC_02867	1	2	21.90	0.623	0.034
Q2FZK9	family transcriptional regulator	SAOUHSC_00992	2	2	16.40	0.650	0.003
Q2G273	urease accessory protein ureg	ureG	3	3	22.30	0.796	0.021
Q2FXX1	acetyl- biotin carboxylase subunit	SAOUHSC_01709	4	4	50.20	0.761	0.022
Q2G1X5	queuosine biosynthesis protein	SAOUHSC_00720	3	3	16.00	0.831	0.000
Q2FXT6	queuine tRNA-ribosyltransferase	tgt	2	2	43.30	0.603	0.008
*Protein synthesis*							
Q2FW17	50s ribosomal protein l24	rplX	2	2	11.50	1.225	0.001
Q2FW29	50s ribosomal protein l36	rpmJ	1	1	4.30	0.704	0.002
Q2FW16	50s ribosomal protein l14	rplN	4	4	13.10	0.734	0.010
Q2FY22	50S ribosomal protein L33 2	rpmG2	1	1	5.90	0.459	0.003
*Stress response*							
Q2FUU5	lipase 1	lipA	2	3	76.60	1.255	0.004
Q2FZS8	chaperone protein clpB	clpB	23	24	98.30	1.211	0.000
Q2G222	N-acetylmuramoyl-l-alanine amidase	SAOUHSC_02979	11	11	69.20	1.259	0.000
Q2G0P6	ATP:guanido phosphotransferase	mcsB	2	2	38.60	1.234	0.010
P72360	iron-sulfur cluster repair di-iron protein	scdA	1	2	25.50	0.796	0.028
Q2G112	single-stranded DNA-binding protein	SAOUHSC_00349	7	7	18.50	0.816	0.014
*Cell wall synthesis and cell division*							
Q2FUW7	accessory sec system glycosylation	gtf1	1	1	58.20	1.591	0.031
Q2G222	N-acetylmuramoyl-l-alanine amidase	SAOUHSC_02979	11	11	69.20	1.259	0.000
P72360	iron-sulfur cluster repair di-iron protein	scdA	1	2	25.50	0.796	0.028
Q2FZ97	transcriptional regulator MraZ	mraZ	3	3	17.20	0.819	0.006
Q2FVQ1	gnat family acetyltransferase	SAOUHSC_02651	1	1	20.10	0.669	0.019
*Membrane permeability and formation*							
Q2FZH1	potassium uptake protein	SAOUHSC_01034	2	2	24.30	1.401	0.037
Q2FZS8	chaperone protein clpB	clpB	23	24	98.30	1.211	0.000
Q2FZD2	Thioredoxin	trxA	9	9	11.40	0.654	0.001
Q2FXI6	Thioredoxin	SAOUHSC_01860	8	8	11.80	0.781	0.003
Q2FXX1	acetyl- biotin carboxylase subunit	SAOUHSC_01709	4	4	50.20	0.761	0.022
*Others*							
Q2FVB3	antibiotic transport system permease	SAOUHSC_02821	1	1	28.90	1.299	0.007
Q2FWL8	transcriptional regulator	SAOUHSC_02271	2	2	8.20	1.448	0.001
Q2G2L6	pf09954 family protein	SAOUHSC_02812	1	1	16.00	1.383	0.019
Q2FZ07	uncharacterized protein	SAOUHSC_01264	2	2	8.20	0.809	0.000
Q2FV28	uncharacterized conserved protein	SAOUHSC_02911	2	2	27.80	0.827	0.023
Q2G2Q9	conserved hypothetical family protein	SAOUHSC_00274	1	2	20.10	0.586	0.001
Q2G297	metal-dependent phosphohydrolase	SAOUHSC_01696	5	5	22.40	0.807	0.002

All accession numbers indicate entries in the UniProt database. *M*w, molecular weight.

**Table 2:** Fatty acid composition of the *Staphylococcus aureus* cell membrane following treatment with juglone for 2 h.

Fatty Acid Species	Control (%)	12.5 μg/mL (%)	25 μg/mL (%)	37.5 μg/mL (%)
C8:0	1.16	1.45	1.31	1.49
C10:0	2.27	2.06	1.97	1.93
C12:0	1.61	1.53	1.45	1.55
C14:0	2.96	2.98	3.06	3.16
C16:1	20.34	21.03	21.56	21.93
C16:0	17.21	17.03	16.52	16.4
C18:2	6.29	6.14	5.62	5.21
C18:1	7.64	7.85	7.92	7.96
C18:0	21.03	20.36	19.14	18.64
UFA	27.98	28.88	29.48	29.89
SFA	38.24	37.39	35.66	35.01
SFA/UFA	1.37	1.3	1.21	1.17

Cn_1_:n_2_, n_1_ and n_2_ represent the number of carbon atoms and olefinic bonds, respectively; SFA, saturated fatty acids, shown as the sum of C16:0 and C18:0; UFA, unsaturated fatty acids, shown as the sum of C16:1 and C18:1.

## Abbreviations

BCA	bicinchoninic acid
FASP	filter-aided proteome preparation
FBD	food-borne diseases
FDR	false discovery rate
GO	gene ontology
HCD	higher-energy collisional dissociation
iTRAQ	isobaric tags for relative and absolute quantitation
LC-ESI-MS/MS	liquid chromatography electrospray ionization tandem mass spectrometry
MDA	malondialdehyde
MIC	minimal inhibition concentration
MS2	second-level mass spectrometry
ROS	reactive oxygen species
SEs	staphylococcal enterotoxins
SFA	saturated fatty acids
SFP	staphylococcal food poisoning
SOD	superoxide dismutase
TAG	triacylglycerols
UFA	unsaturated fatty acids
WHO	World Health Organization
GTP	guanosine triphosphate
CTP	cytidine triphosphate
CAT	catalase
